# Dynamic portfolio choice with uncertain rare-events risk in stock and cryptocurrency markets

**DOI:** 10.1186/s40854-023-00472-8

**Published:** 2023-04-05

**Authors:** Wujun Lv, Tao Pang, Xiaobao Xia, Jingzhou Yan

**Affiliations:** 1 Department of Statistics, College of Science, Donghua University, No. 2999 Renmin North Road, Songjiang District, Shanghai, 201620 China; 2 Department of Mathematics, North Carolina State University, 2311 Stinson Drive, Raleigh, NC 27695-8205 USA; 3 School of Economics, Fudan University, 600 Guoquan Road, Shanghai, 200433 China; 4 School of Economics, Sichuan University, 24 South Section 1, Yihuan Road, Chengdu, 610065 Sichuan China

**Keywords:** Robust portfolio choice, Detection error probability, Rare events, Ambiguity, Cryptocurrency, Welfare loss, D81, G11, G41

## Abstract

In response to the unprecedented uncertain rare events of the last decade, we derive an optimal portfolio choice problem in a semi-closed form by integrating price diffusion ambiguity, volatility diffusion ambiguity, and jump ambiguity occurring in the traditional stock market and the cryptocurrency market into a single framework. We reach the following conclusions in both markets: first, price diffusion and jump ambiguity mainly determine detection-error probability; second, optimal choice is more significantly affected by price diffusion ambiguity than by jump ambiguity, and trivially affected by volatility diffusion ambiguity. In addition, investors tend to be more aggressive in a stable market than in a volatile one. Next, given a larger volatility jump size, investors tend to increase their portfolio during downward price jumps and decrease it during upward price jumps. Finally, the welfare loss caused by price diffusion ambiguity is more pronounced than that caused by jump ambiguity in an incomplete market. These findings enrich the extant literature on effects of ambiguity on the traditional stock market and the evolving cryptocurrency market. The results have implications for both investors and regulators.

## Introduction

The last decade witnessed unprecedented economic uncertainty in the financial market. Episodes of uncertainty include the subprime crisis of 2008, Brexit in 2016, and the Sino-US trade conflict in 2019. The most recent and striking event is exemplified by the COVID-19 pandemic, which began in January 2020 and exerted a tremendous impact on the global economy (Altig et al. 2020) in the last 2 years. According to the Wall Street Journal, on March 9, 2020, S &P 500 fell by 7% within several minutes, triggering a circuit breaker. Then in the second quarter in 2020, S &P 500 Index rose by 19.95%, the DJIA rose by 17.77%, and the Nasdaq Index rose by 30.63%, with huge volatility. These rare events that precipitate a sudden shock to both security prices and volatilities are prevalent in the financial market. Examples include the 1987 stock crash and the 1997 Long Term Capital Market (LTCM) collapse (Liu et al. 2003; Cheng and Yang 2017). Such rare events in the stock market naturally cause investors to feel anxious about their investments, especially those in emerging economies with limited access to different financial derivatives.^1^ Furthermore, the burgeoning cryptocurrency market is extremely highly volatile (Xu et al. 2019). Nobel Prize laureate Robert Shiller also highlighted that the source of value of Bitcoin is exceptionally ambiguous.^2^ Cryptocurrency markets experienced exponential growth in 2017 and a bubble burst in early 2018. In the wake of the pandemic, the market value of cryptocurrencies rose considerably in 2020. The market value of cryptocurrencies has been very volatile but consistently at historically high levels in 2021 (Fang et al. 2022). These facts naturally make optimal portfolio allocation a novel issue. Therefore, we examine optimal portfolio choice under ambiguity in these two markets, when investors are uncertain about rare events.

The motivation behind this study is to recognize the dichotomy between ambiguity and the rare events model in the current literature that has three strands. The first strand examines rare events without considering the inherent ambiguity, and is exemplified by the seminal Duffie et al. (2000), Liu et al. (2003), and Bates (2019) using stochastic volatility model with multi-jump (SVMJ) framework to study stock market problems. The second strand discusses only jump ambiguity and is represented in studies by Jin et al. (2018), Aït-Sahalia and Matthys (2019), and Jin et al. (2021). The third strand reflected in studies such as Branger and Larsen (2013) and Drechsler (2013), considers different ambiguities, including price diffusion and jump ambiguity, without focusing on rare events. Hence, we introduce ambiguity into the traditional stock market and cryptocurrency market models with rare events to capture such events more accurately. We answer the following questions: How can ambiguity influence the detection-error probability (DEP), measuring the ambiguity between the two models?^3^ Which effects do different ambiguities exert on the investors’ portfolio choice? Do downward jumps (negative price jumps) and upward jumps (positive price jumps) have an asymmetric effect? What is the effect of different ambiguities on welfare loss? Are the effects on optimal portfolio choice in the traditional stock market and the cryptocurrency market different?

The main findings of this study are summarized as follows. First, we derive a semi-closed form solution for the optimal portfolio choice and DEP with uncertain rare events risks. In particular, DEP is determined by price diffusion and jump. Second, optimal portfolio choice is more significantly affected by price diffusion ambiguity than by jump ambiguity, and the effect of the volatility diffusion ambiguity is trivial. Third, we find an asymmetric effect on portfolio choice and explain this effect from the perspective of the disposition effect.^4^ For negative price jumps, investors “speculate” by increasing the exposure. Intuitively, if they are ambiguity averse to a series of negative stock jumps, then increase in the volatility jump size decreases their probability of earning successive negative returns and increases the probability of market reversal. By contrast, they “de-risk” their positions by decreasing the exposure to upward price jumps. Therefore, our model can explain the “disposition effect,” widely acknowledged as one of the most robust features about trading by individual investors (Barberis and Xiong 2009) in behavioral finance.^5^ Finally, welfare loss caused by price diffusion ambiguity and jump ambiguity is more significant.

Based on the main results, our study has policy implications in two aspects. First, investors should decrease portfolio choice, decrease leverage, and seek more information^6^ when facing high market ambiguity aversion. Second, regulators are responsible for educating the investors, developing diverse financial market products, and strengthening reasonable supervision.

Our study is related to ambiguity aversion in stock market literature. Anderson et al. (2003) set the theoretical cornerstone for the following research by developing a robust approach in a continuous-time setting to penalize investors’ belief distortions from reference probability. Maenhout (2004, 2006) use this approach to derive portfolio choice rules reduced to uncertainty about the drift of state variables. Researchers examine the optimal portfolio choice problem under ambiguity from different perspectives: Branger and Larsen (2013) consider price diffusion and jump risk. Aït-Sahalia and Matthys (2019) consider price diffusion ambiguity and jump ambiguity. Jin et al. (2021) discuss ambiguous jumps with arbitrary tail assumptions. Yan et al. (2020) introduce uncertainty to drift in the prices of risky assets and the covariance matrix of asset returns. Yang et al. (2020) analytically examine a multi-factor volatility model. Cheng and Escobar-Anel (2021) allow separate levels of uncertainty for variance. Our study incorporates price diffusion, volatility diffusion, and jump ambiguity into a single framework. In addition, Agliardi (2018) explores ambiguity in the calculation of Value-at-Risk in terms of capital requirement.

Our study is also related to financial risk. Theoretically, Liu et al. (2003) consider an optimal portfolio choice model with stochastic volatility and jumps. Liu and Pan (2003) extend the above study to the case of a complete market. Branger et al. (2008) investigate the consequences of incorrectly including or omitting jumps in volatility. Chen et al. (2017) discuss the asset allocation under loss aversion in a defined-contribution pension plan. Mu et al. (2020) investigate the effects of jump risk on fund managers’ optimal portfolio choice under a high-water mark contract. Empirically, Kou et al. (2014) apply machine learning methods to assess financial risks. Kou et al. (2019) clarify the research methods in current cutting-edge machine learning technology to assess and measure financial risks.

Our study is also related to the cryptocurrency market. Sebastião and Godinho (2021) examine the predictability of three major cryptocurrencies with machine learning methods. Hou et al. (2020) study the pricing model of cryptocurrency options. Huang et al. (2022) study the leverage effect in cryptocurrency markets.

The remainder of the paper is organized as follows. In " Model setup" section, we formulate the problem and set up the model. In "Main results" section, we derive the verification theorem, solutions of optimal portfolio choice in the stock market, detection error probability, and welfare loss. In "Numerical results and discussions" section, we conduct numerical analysis. In “Cryptocurrency market” section, we analyze the optimal portfolio choice in the cryptocurrency market. In “Conclusion” section, we conclude the paper. All proofs are provided in the Appendix.

## Model setup

### The rare-events risk model

In this section, we focus on dynamic portfolio choice with uncertain rare event risk in the stock market. We consider an investor who has two investment choices. The first is a risk-free asset $M_t$ in the money market evolving according to $dM_{t}=rM_{t}dt$, where *r* is a fixed constant interest rate. The second is a risky asset, for instance, a stock, with the price $S_t$ process evolving according to

1 $$\begin{aligned} dS_{t}=(r+\eta V_t )S_t dt + \sqrt{V_t} S_t d{\mathcal{Z}}_{1,t} + S_{t} (X_t d{N}_t- \mu \lambda V_t dt), \end{aligned}$$

and the instantaneous volatility $V_t$ of a given cryptocurrency follows a diffusion-jump process

2 $$\begin{aligned} {dV_{t}}=( \alpha - \beta V_t )dt + \sigma \sqrt{V_t} (\rho d{\mathcal{Z}}_{1,t} +\sqrt{1-\rho ^2} d{\mathcal{Z}}_{2,t}) + Y_t d{N}_t -\kappa \lambda V_t dt, \end{aligned}$$

as per Bates (2000), Pan (2002), Liu et al. (2003) and Bates (2019), $\eta V_t$ is a risk premium and $\mu \lambda V_t S_t$ can be interpreted as the compensation for expected return due to jump risk of the stock price. Similarly, $\kappa \lambda V_t$ can be treated as compensation due to the jump risk of volatility. ${\mathcal{Z}}_{1,t}$ and ${\mathcal{Z}}_{2,t}$ are two standard, independent Brownian motions depicting price diffusion and volatility diffusion under the reference measure ${\mathbb{P}}$. ${N_t}$ is a Poisson process independent of ${\mathcal{Z}}_{1,t}$ and ${\mathcal{Z}}_{2,t}$ with stochastic arrival intensity $\lambda V_t$ (Bates 2000, 2019). $\rho$ is the correlation coefficient of two Brownian motions driving $S_t$ and $V_t$. All parameters $\alpha , \beta , \lambda , \kappa$, and $\sigma$ are nonnegative. $X_t$ and $Y_t$ are jump sizes that characterize price jumps and volatility jumps, respectively; they are assumed to be independent of ${\mathcal{Z}}_{1,t},{\mathcal{Z}}_{2,t}$, and ${N_t}$. To ensure that $S_t$ is positive (limited liability), the price jump size is $X_t$ with mean $\mu ,$ and support on $(-1,\infty )$. Similarly, to ensure that $V_t$ is positive, the volatility jump size is $Y_t$ with mean $\kappa$, and support on $[0,\infty )$.

### Ambiguity aversion and optimal portfolio problem

Now we assume that the investor is ambiguous about the model given by (1) and (2), which governs the evolution of stock price and instantaneous volatility of stock price, due to uncertainty about small diffusion risk as well as large rare-event risk shocks. We further assume that though the investor uses the model given by (1) and (2) as their reference model, they are skeptical about this model. The investor will consider alternative models defined by probability measures. We use $\widetilde{{\mathbb{P}}}$ to denote an alternative probability measure. Following Branger and Larsen (2013), Drechsler (2013) and Mao et al. (2022), we assume that each alternative measure $\widetilde{{\mathbb{P}}}$ is defined by the Radon-Nikodym derivative

$$\begin{aligned} \begin{aligned} \Lambda _{t}=\exp \left( {- \int _0^t u^S_s d{\mathcal{Z}}_{1,s} - \frac{1}{2} \int _0 ^t (u^S_s)^2 ds} {- \int _0^t u^V_s d{\mathcal{Z}}_{2,s} - \frac{1}{2} \int _0 ^t (u^V_s)^2 ds + \int _0 ^t \ln u^N_s d{N}_s+ \int _0 ^t (1-u^N_s) \lambda V ds} \right) \end{aligned} \end{aligned}$$

is a $\left( {\mathbb{P}}, {\mathscr {F}} \right)$ martingale with $\Lambda _{ 0}=1$. By Ito’s formula, we get the following SDE

3 $$\begin{aligned} \frac{d\Lambda _{t}}{\Lambda _{t}}=&{- u^S_t d{\mathcal{Z}}_{1,t} } {- u^V_t d{\mathcal{Z}}_{2,t} } +(u^N_t-1) d{N}_t - (u^N_t-1) \lambda V_t dt. \end{aligned}$$

By Girsanov’s Theorem, under the alternative measure $\widetilde{{\mathbb{P}}}$, the processes $\widetilde{{\mathcal{Z}}}_{1,t}$ and $\widetilde{{\mathcal{Z}}}_{2,t}$ defined by $d\widetilde{{\mathcal{Z}}}_{1,t} = d{{\mathcal{Z}}}_{1,t} +u^S_t dt, d\widetilde{{\mathcal{Z}}}_{2,t}= d{{\mathcal{Z}}}_{2,t} +u^V_t dt$ are two standard Brownian motions, ${N_t}$ is a Poisson process; the corresponding jump intensity changes from $\lambda V_t$ to $u^{N} \lambda V_t$, and $u^{N}$ is a perturbation parameter or scaled term (Drechsler 2013). For convenience, let $\widetilde{N}_t$ represent the price and volatility jump risk under alternative measure $\widetilde{{\mathbb{P}}}$. Furthermore, the stock price and instantaneous volatility under $\widetilde{{\mathbb{P}}}$ can be characterized by the following equation

4 $$\begin{aligned} dS_{t} & {} = (r+\eta V_t -\sqrt{V_t} u^S_t - \mu V_t \lambda )S_t dt + \sqrt{V_t} S_t d\widetilde{{\mathcal{Z}}}_{1,t}+ S_{t-} X_t d\widetilde{{N}}_t,\nonumber \\{} {dV_{t}} & {}=( \alpha - \beta V_t - \sigma \rho \sqrt{V_t} u^S_t -\sigma \sqrt{1-\rho ^2} \sqrt{V_t} u^V_t - \kappa \lambda V_t ) dt \nonumber \\{} & {} + \sigma \sqrt{V_t} (\rho d\widetilde{{\mathcal{Z}}}_{1,t} +\sqrt{1-\rho ^2} d\widetilde{{\mathcal{Z}}}_{2,t})+Y_t d\widetilde{{N}}_t. \end{aligned}$$

Now, we define $W_t$ as the investor’s wealth. The fraction $\pi _t$ of the wealth $W_t$ is invested in a risky asset, while the remaining $(1-\pi _t) W_t$ is invested in a risk-free asset. Its dynamic under the alternative measure $\widetilde{{\mathbb{P}}}$, evolves as follows:

5 $$\begin{aligned} \begin{aligned} dW_{t} =&{(r+\pi _t (\eta V_t - \mu V_t \lambda - \sqrt{V_t} u^S_t ) ) W_t}dt + \pi _t \sqrt{V_t} W_t d\widetilde{{\mathcal{Z}}}_{1,t}+ \pi _t X_t W_{t} d\widetilde{{N}}_t . \end{aligned} \end{aligned}$$

Following Anderson et al. (2003), Hansen et al. (2006), and Jin et al. (2021), we assume that the investor seeks an optimal robust portfolio choice which is the optimal portfolio decision in some worst-case models. Thus, under the alternative measure $\widetilde{{\mathbb{P}}}$, we define the investor’s robust indirect utility function as

6 $$\begin{aligned} J(t,w,v)= \sup _{\pi } \inf _{u^S, u^V, u^N} {\mathbb{E}}^{\widetilde{{\mathbb{P}}}}_{t,w,v} \left[ U(W_T) +\int _t^T \Psi (s,w,v) ds \right] , \end{aligned}$$

where,

7 $$\begin{aligned} U(w) & {} = \frac{w^{1-\gamma }}{1-\gamma },\nonumber \\{} \Psi (t,w,v) & {} = \frac{(u^S_{t})^2}{2 \Psi ^S(t,w,v)} + \frac{(u^V_{t})^2}{2 \Psi ^V(t,w,v)}+ \lambda v \frac{u^N_{t} \ln u^N_{t} -u^N_{t} +1}{\Psi ^N(t,w,v)}, \end{aligned}$$

where the expectation on the right-hand side of Eq. (6) is calculated under the alternative measure ${\widetilde{{\mathbb{P}}}}$ defined by ${u^S, u^V}$ and $u^N$ to account for uncertain rare events. The investor chooses $u^S_{t}, u^V_{t}$, and $u^N_{t}$ by considering the worst case, and lets $u = (u^S,u^V,u^N)$, ${\mathbb{E}}^{\widetilde{{\mathbb{P}}}}_{t,w,v}[\cdot ] = {\mathbb{E}}^{\widetilde{{\mathbb{P}}}}_{t,w,v}[\cdot | (W_t,V_t)=(w,v)]$. The first term in the indirect utility function, $U(W_t) = \frac{W^{1-\gamma }_t}{1-\gamma }$, is a power utility function used in literature, and $\gamma > 0$ is the coefficient of relative risk aversion. The second term in the indirect utility function, ${\mathbb{E}}^{\widetilde{{\mathbb{P}}}}_t \left[ \int _t^T \Psi (s,w,v) ds \right]$, is a new term to penalize model uncertainty deviation from the reference probability measure. This penalty term depends on the relative entropy arising from price diffusion, volatility diffusion, and jump ambiguity. Specifically, according to Branger and Larsen (2013), Drechsler (2013), and Jin et al. (2021), we obtain the relative entropy over the time interval $[t, t + \Delta t]$ to measure discrepancy between ${\mathbb{P}}$ and ${\widetilde{{\mathbb{P}}}}$

8 $$\begin{aligned} 
{\mathbb{E}}_t^{\widetilde{{\mathbb{P}}}} \left[ \ln \left( \frac{ \Lambda _{t+ \Delta t}}{\Lambda _{t}}\right) \right] & {} = {\mathbb{E}}_t^{\widetilde{{\mathbb{P}}}} \left[ \int _t^{t + \Delta t} \frac{1}{2 } (u^S_{s})^2 + \frac{1}{2 } (u^V_{s})^2 + \lambda v ({u^N_{s} \ln u^N_{s} -u^N_{s} +1}) ds \right] \nonumber \\{} & {}\overset{\Delta t \rightarrow 0}{\longrightarrow } \frac{1}{2 } (u^S_{t})^2 + \frac{1}{2 } (u^V_{t})^2 + \lambda v ({u^N_{t} \ln u^N_{t} -u^N_{t} +1}). \end{aligned}$$

In the penalty term $\Psi (t,w, v)$, the three terms of Eq. (8) are scaled by $\Psi ^S, \Psi ^V$, and $\Psi ^N$. This scaling captures investor ambiguity aversion with respect to price diffusion, volatility diffusion, and jump risk. Clearly, the larger the $\Psi ^S, \Psi ^V$ and $\Psi ^N$, the higher the ambiguity aversion and thus, the smaller the penalty for deviating from the reference model.

Let $\Pi$ denote the set of all the portfolio choices, taking values in *R*. Let $\Omega$ denote the set of all the worst-case measures, taking values in $R^3$. Next, we define admissibility of a portfolio choice as follows.

#### Definition 1

A portfolio choice (or control) $\pi _t$ is said to be admissible, $\pi \in \Pi$, if $\pi (t)$ is a progressively measurable process with respect to filtration ${\mathcal{F}}_t$ such that a unique solution to Eq. (5) exists and ${\mathbb{E}}^{\widetilde{{\mathbb{P}}}}_{t,w,v} \left[ U(W_T) +\int _t^T \Psi (s,w,v) ds \right] < \infty$, for all $u \in \Omega$, $(s,w,v) \in [0,T] \times R^2$.

## Main results

In this section, we present our main results on the verification theorem, optimal portfolio choice, detection error probability (DEP), and welfare loss.

### The optimal robust portfolio choice

For convenience, we introduce some notations. Let

$$\begin{aligned} C^{1,2,2}([0,T) \times R^2)&= \{J(t,w,v)|J(t,\cdot ,\cdot ) \ be \ once \ continuously \ differentiable \ on \ [0,T] \\&\ and \ J(\cdot ,w,v) \ be \ twice \ continuously \ differentiable \ on \ R^2\}. \end{aligned}$$

For any $(t,w,v) \in ([0,T) \times R^2)$ and $J(t,w,v) \in C^{1,2,2}([0,T) \times R^2)$, we define an infinitesimal generator as

9 $$\begin{aligned} {\mathcal{L}}^{u,\pi } J(t,w,v)= & {} J_t + r w J_w + \pi (\eta v - \mu \lambda v- \sqrt{v} u^S ) w J_w + \frac{1}{2} v w^2 \pi ^2 J_{ww} \nonumber \\{} & {} \quad + \frac{1}{2} \sigma ^2 v J_{vv}+ ( \alpha - \beta v -\kappa \lambda v - \sigma \rho \sqrt{v} u^S -\sigma \sqrt{1-\rho ^2} \sqrt{v} u^V )J_v \nonumber \\{} & {} \quad + \rho \pi \sigma v w J_{wv} + \lambda v u^N {\mathbb{E}} \left[ J(t, w(1+\pi X),v +Y)-J \right] \,, \end{aligned}$$

where $J_{t},$ $J_{v},$ $J_{w},$ $J_{vv},$ $J_{ww}$, and $J_{wv}$ denote the first and second derivatives with respect to *t*,  *w*, and *v*, and the expectation is taken as a joint distribution of *X* and *Y*.

We first solve the optimization problem (6) subject to constraints (4) and (5). As per stochastic dynamic programming mentioned in Anderson et al. (2003), Hansen et al. (2006), Jin et al. (2021), and Xu et al. (2022), the investor’s robust indirect utility function *J* satisfies the following Hamilton-Jacobi-Bellman-Isaacs (HJBI) equation:

10 $$\begin{aligned} 0= & {} \sup _{\pi \in \Pi } \inf _{u \in U} {\mathcal{L}}^{u,\pi } J(t,w,v) + \Psi \,. \end{aligned}$$

For tractability, we assume $\Psi ^S$, $\Psi ^V$, and $\Psi ^N$ are state-dependent. Following Maenhout (2004, 2006) and Luo et al. (2021b), we set

11 $$\begin{aligned} \Psi ^i(t,w,v) = \frac{\theta _i}{(1-\gamma )J (t,w,v)}, i \in \{S, V, N \}, \end{aligned}$$

where ambiguity aversion with respect to price diffusion, volatility diffusion, and jump risk is increasing in the parameters $\theta _S,$ $\theta _V,$ and $\theta _N$, respectively.

The following proposition shows the conditions under which the solution to the HJBI equation is the value function, and the control is the optimal robust portfolio choice.

#### Proposition 1

(Verification Theorem). If there exists a function $J \in C^{1,2,2}([0,T) \times R^2) \cap C([0,T) \times R^2)$ and an optimal control $(u^*,\pi ^*) \in \Omega \times \Pi$ such that

(i) ${\mathcal{L}}^{u,\pi ^*} J(t,w,v)+ \Psi (s,w,v,u,\pi ^*) \ge 0$, for all $u \in \Omega$ and $(w,v) \in R^2$,
(ii) ${\mathcal{L}}^{u^*,\pi } J(t,w,v)+ \Psi (s,w,v,u^*,\pi ) \le 0$, for all $\pi \in \Pi$ and $(w,v) \in R^2$,
(iii) ${\mathcal{L}}^{u^*,\pi ^*} J(t,w,v)+ \Psi (s,w,v,u^*,\pi ^*) = 0$, for all $u \in \Omega$ and $(w,v) \in R^2$,
(iv) $J(T,w,v) = U(w_T)$ for all $\pi \in \Pi$, $u \in \Omega$ and $(t,w,v) \in [0,T)\times R^2$ and,
(v) the family $\{J(\tau ,W(\tau ), V(\tau )) \}_{\tau \in {\mathcal{T}}}$ is uniformly integrable, where ${\mathcal{T}}$ denotes the set of stopping times $\tau \le T$, for all $(u,\pi ) \in \Omega \times \Pi$, and $(t,w,v) \in [0,T)\times R^2$.

Then,

$$\begin{aligned} \inf _{u \in \Omega } \sup _{\pi \in \Pi } O^{u,\pi } (t,w,v) \le J(t,w,v) \le \sup _{\pi \in \Pi }\inf _{u \in \Omega } O^{u,\pi }(t,w,v). \end{aligned}$$

Indeed,

$$\begin{aligned} J(t,w,v) = \inf _{u \in \Omega } \sup _{\pi \in \Pi } O^{u,\pi } (t,w,v) = \sup _{\pi \in \Pi } \inf _{u \in \Omega } O^{u,\pi }(t,w,v), \end{aligned}$$

and $(u^*,\pi ^*)$ is an optimal control, and the objective function is

12 $$\begin{aligned} O^{u,\pi } (t,w,v) = {\mathbb{E}}^{\widetilde{{\mathbb{P}}}}_{t,w,v} \left[ U(W_T) +\int _t^T \Psi (s,W_s,V_s, u_s,\pi _s) ds \right] . \end{aligned}$$

Next, the following proposition gives an analytical solution for the indirect utility function, optimal robust portfolio choice, and the worst-case measure.

#### Proposition 2

The indirect utility function is in the form given by

13 $$\begin{aligned} J(t,w,v)=\frac{w^{1-\gamma }}{1-\gamma } e^{A(t)+B(t)v}, \end{aligned}$$

the optimal robust portfolio choice is given by

14 $$\begin{aligned} \begin{aligned} \pi ^* = \frac{1}{ \theta _S+\gamma } \left( (\eta -\mu \lambda ) + \left( 1- \frac{ \theta _S }{ 1-\gamma }\right) \rho \sigma B(t) + {\lambda { {\mathbb{E}} [ (1+\pi ^* X)^{-\gamma } X e^{B(t)Y} ]}} u^{N*}\right) , \end{aligned} \end{aligned}$$

and the worst-case measure is given by

15 $$\begin{aligned} u^{S*}= & {} (\pi ^* + \frac{1}{1-\gamma } \sigma \rho B(t)) \theta _S \sqrt{v}\,, \end{aligned}$$

16 $$\begin{aligned} u^{V*}= & {} \frac{1}{1-\gamma } \sigma \sqrt{1-\rho ^2} B(t) \theta _V \sqrt{v}\,, \end{aligned}$$

17 $$\begin{aligned} u^{N*}= & {} e^{ - \frac{\theta _N}{1-\gamma } \left( {\mathbb{E}} [ (1+\pi ^* X)^{1-\gamma } e^{B(t)Y}]-1 \right) }\,, \end{aligned}$$

where *B*(*t*) and *A*(*t*) satisfy the following differential equation

18 $$\begin{aligned} 0= & {} \frac{1}{1-\gamma }B^{\prime }(t) +\pi ^{*} ( \eta - \mu \lambda )- \frac{1}{2} \pi ^{*2} (\gamma + \theta _S) +\left( \frac{1 }{2(1-\gamma )} - \frac{ \rho ^2 \theta _S + (1-\rho ^2) \theta _V}{ 2(1-\gamma )^2}\right) \sigma ^2 B^2(t) \nonumber \\{} & {} \quad + \left( \rho \sigma \pi ^{*}+ \frac{\pi ^{*} \sigma \rho \theta _S - (\beta +\kappa \lambda ) }{1-\gamma } \right) B(t) + \lambda \frac{1}{\theta _N} \left( 1- e^{ - \frac{\theta _N}{1-\gamma } \left( {\mathbb{E}} [ (1+\pi ^* X)^{1-\gamma } e^{B(t)Y}]-1 \right) } \right) , \end{aligned}$$

and

19 $$\begin{aligned} 0=&r (1-\gamma ) + A^{\prime }(t) + B(t) \alpha . \, \end{aligned}$$

In addition, $$J(T,w,v) = \frac{w^{1-\gamma }_T}{1-\gamma }$$.

#### Proof

See Appendix B.

The optimal portfolio choice in (14) consists of three terms. The first term is instantaneous risk premium divided by the risk aversion parameter plus ambiguity aversion to price diffusion. When $\lambda$ is 0 and $V_t$ is not stochastic, it reduces to $\frac{\eta }{ \theta _S+\gamma }$, similar to the usual myopic component. But here $\theta _S$ plays an important role because the larger the $\theta _S$, the smaller the optimal portfolio choice. The second term is related to the correlation coefficient between instantaneous return and change of volatility. The third term is directly related to two parts: the first part is ${\mathbb{E}} [ (1+\pi ^* X)^{-\gamma } X e^{B(t)Y} ]$, representing a blend of dynamic portfolio choice and static buy-and-hold portfolio choice, which is called M1 in Liu et al. (2003), and the second part is $u^{N*}$, representing the worst-case measure about the jump risk in (17).

### The detection-error-probability (DEP)

We estimate the parameters that characterize the preference for ambiguity aversion, $\theta _S, \theta _V,$ and $\theta _N$ based on DEP, $\epsilon _T(\theta _S, \theta _V, \theta _N)$. According to Anderson et al. (2003), Maenhout (2006), and Aït-Sahalia and Matthys (2019), $\theta _S, \theta _V,$ and $\theta _N$ should be chosen in such a way that it is difficult to distinguish the reference model from the worst-case model. Since our robust portfolio choice depends on $\theta _S, \theta _V,$ and $\theta _N$, we accordingly define DEP, denoted by $\epsilon _T(\theta _S, \theta _V, \theta _N)$, at time zero as

20 $$\begin{aligned} \epsilon _T(\theta _S, \theta _V, \theta _N) = \frac{1}{2} \Pr (\xi _{T}>0| {\mathbb{P}}, {\mathscr {F}}_0) + \frac{1}{2} \Pr (\xi _{T}<0| \widetilde{{\mathbb{P}}}, {\mathscr {F}}_0), \end{aligned}$$

where $\xi _{t} \equiv \ln \Lambda _{t}$. Given a finite time series, the investor’s decision reduces to two cases: accidentally discarding the reference model for the worst-case model (model $\widetilde{{\mathbb{P}}}$ ) if $\xi _{T}>0$ or rejecting the worst-case model erroneously if $\xi _{T}<0$. If the difference between the two models is large, it is easy to distinguish these two models from each other, and DEP is small, and vice versa. As recommended by Anderson et al. (2003) and Maenhout (2006), we will use ${\theta _{{S}}}$, $\theta _V$, and ${\theta _N}$ to ensure that $\epsilon _T(\theta _S$ , $\theta _V$, $\theta _N)$ is at least 10%.

Then, the two conditional probabilities in $\epsilon _T(\theta _S, \theta _V, \theta _N)$ can be obtained by finding the conditional characteristic functions of $\xi _{T}$ under ${\mathbb{P}}$ and $\tilde{{\mathbb{P}}}$, denoted by $\phi _{{\mathbb{P}}}(\omega ,t,T)$ and $\phi _{\widetilde{{\mathbb{P}}}}(\omega ,t,T)$, respectively, where $\omega$ is the usual transform variable. In particular, $\phi _{{\mathbb{P}}}(\omega ,t,T)$ and $\phi _{\widetilde{{\mathbb{P}}}}(\omega ,t,T)$ are defined as the Fourier transforms of the conditional expectation

21 $$\begin{aligned} \begin{aligned} \phi _{{\mathbb{P}}}(\omega ,t,T) = {\mathbb{E}}{^{\mathbb{P}}}[e^{i \omega \xi _{T}}|{\mathscr {F}}_t ]= {\mathbb{E}}{^{\mathbb{P}}}[{\Lambda ^{i \omega }_{T} }|{\mathscr {F}}_t ], \end{aligned} \end{aligned}$$

where $i=\sqrt{-1}$ is an imaginary unit. We use simple measure change of the form

22 $$\begin{aligned} \begin{aligned} \phi _{\widetilde{{\mathbb{P}}}}(\omega ,t,T) = {\mathbb{E}}^{\widetilde{{\mathbb{P}}}} [{\Lambda ^{i \omega }_{T} }|{\mathscr {F}}_t ]= {\mathbb{E}}{^{\mathbb{P}}}[e^{i \omega \xi _{T}} e^{\xi _{T}}|{\mathscr {F}}_t ]= {\mathbb{E}}{^{\mathbb{P}}}[{\Lambda ^{i \omega +1}_{T} }|{\mathscr {F}}_t ], \end{aligned} \end{aligned}$$

and we define $\varvec{u}=[u^S, u^V]^T$ and $\varvec{\sigma } = [\rho \sigma \sqrt{V}, \sigma \sqrt{V} \sqrt{1-\rho ^2}]^T$. On applying the Feynman-Kac theorem, $\phi _{{\mathbb{P}}}(\omega , t, T)$ and $\phi _{\widetilde{{\mathbb{P}}}}(\omega , t, T)$ are given by the following differential equation:

23 $$\begin{aligned} \begin{aligned} 0 =&\frac{ \partial \phi _{{\mathbb{P}}} }{\partial t} + (\alpha - \beta V - \kappa \lambda V) \frac{ \partial \phi _{{\mathbb{P}}} }{\partial V} -\frac{ \partial \phi _{{\mathbb{P}}} }{\partial \Lambda } \Lambda (u^N -1 )\lambda V +\frac{1}{2} \frac{ \partial ^2 \phi _{{\mathbb{P}}} }{\partial \Lambda ^2} \Lambda ^2 \Vert \varvec{u} \Vert ^2 +\frac{1}{2} \sigma ^2 V \frac{ \partial ^2 \phi _{{\mathbb{P}}} }{\partial V^2}\\&- \frac{ \partial ^2 \phi _{{\mathbb{P}}} }{\partial \Lambda \partial V} \Lambda \varvec{u} \varvec{\sigma }^T + \lambda V {\mathbb{E}} \left[ \phi _{{\mathbb{P}}} ( \omega , t, T)-\phi _{{\mathbb{P}}} (\omega , t-, T) \right] , \end{aligned} \end{aligned}$$

24 $$\begin{aligned} \begin{aligned} 0 =&\frac{ \partial \phi _{\widetilde{{\mathbb{P}}}} }{\partial t} +(\alpha - \beta V - \kappa \lambda V ) \frac{ \partial \phi _{\widetilde{{\mathbb{P}}}} }{\partial V} -\frac{ \partial \phi _{\widetilde{{\mathbb{P}}}} }{\partial \Lambda } \Lambda (u^N -1 )\lambda V +\frac{1}{2} \frac{ \partial ^2 \phi _{\widetilde{{\mathbb{P}}}} }{\partial \Lambda ^2} \Lambda ^2 \Vert \varvec{u} \Vert ^2 +\frac{1}{2} \sigma ^2 V \frac{ \partial ^2 \phi _{\widetilde{{\mathbb{P}}}}}{\partial V^2} \\&- \frac{ \partial ^2 \phi _{\widetilde{{\mathbb{P}}}} }{\partial \Lambda \partial V} \Lambda \varvec{u} \varvec{\sigma }^T + \lambda V {\mathbb{E}} \left[ \phi _{\widetilde{{\mathbb{P}}}} ( \omega , t, T)-\phi _{\widetilde{{\mathbb{P}}}} (\omega , t-, T) \right] , \end{aligned} \end{aligned}$$

where $\Vert \cdot \Vert$ is the $L^2$ norm, and $\phi _{{\mathbb{P}}}(\omega ,T,T) = \Lambda ^{i \omega }_{T}$ and $\phi _{\widetilde{{\mathbb{P}}}}(\omega ,T,T) = \Lambda ^{i \omega +1}_{T}$ are boundary conditions.

Applying Lévy’s general inversion, we obtain DEP, $\epsilon _T(\theta _S, \theta _V, \theta _N)$ as the following:

25 $$\begin{aligned} \epsilon _T(\theta _S, \theta _V, \theta _N) = \frac{1}{2} - \frac{1}{2 \pi } \int _0^\infty \left\{ \Re \left( \frac{\phi _{\widetilde{{\mathbb{P}}}}(\omega , 0, T)}{i \omega } \right) -\Re \left( \frac{\phi _{{\mathbb{P}}}(\omega , 0, T)}{i \omega } \right) \right\} d \omega . \end{aligned}$$

The following proposition summarizes how to compute DEP in a semi-closed form.

#### Proposition 3

1. . Solution to the differential Eq. (23) is given by
  26 $$\begin{aligned} \phi _{{\mathbb{P}}}(\omega ,T,t)=\Lambda ^{i \omega }_{t}e^{D(t)+E(t)V}, \end{aligned}$$
  where *D*(*t*) and *E*(*t*) satisfy the differential equation system given by
  27 $$\begin{aligned} 0= & {} D'(t) + \alpha E(t), \end{aligned}$$
  28 $$\begin{aligned} 0= & {} E'(t) - (\kappa \lambda + \beta ) E(t) - i \omega (u^N -1 )\lambda + \frac{1}{2} i \omega (i \omega -1) ((p^S)^2 +(p^V)^2) \nonumber \\{} & {} \quad +\frac{1}{2} \sigma ^2 E^2(t)- E(t) i \omega (\sigma \sqrt{1-\rho ^2} p^V +\rho \sigma p^S ) + \lambda {\mathbb{E}} \left[ (u^N)^{i \omega } e^{E(t)Y}-1 \right] . \end{aligned}$$
2. . Solution to the differential Eq. (24) is given by
  29 $$\begin{aligned} \phi _{\widetilde{{\mathbb{P}}}}(\omega ,t,T) = \Lambda ^{i \omega +1}_{t}e^{F(t)+H(t)V}, \end{aligned}$$
  where *F*(*t*) and *H*(*t*) satisfy the differential equation system given by
  30 $$\begin{aligned} 0= & {} F'(t) + \alpha H(t), \end{aligned}$$
  31 $$\begin{aligned} 0= & {} H'(t) - (\kappa \lambda + \beta ) H(t) - (i \omega +1) (u^N -1 )\lambda + \frac{1}{2} i \omega (i \omega +1) ((p^S)^2 +(p^V)^2) \nonumber \\{} & {} \quad +\frac{1}{2} \sigma ^2 H^2(t) - H(t) (i \omega +1) (\sigma \sqrt{1-\rho ^2} p^V +\rho \sigma p^S ) + \lambda {\mathbb{E}} \left[ (u^N)^{i \omega +1} e^{H(t)Y}-1 \right] , \end{aligned}$$
  where $$p^S = {u^S}/{\sqrt{V}}$$, $$p^V = {u^V}/{\sqrt{V}}$$.

#### Proof

See Appendix C.

### Welfare loss

In this subsection, we gauge investors’ welfare losses stemming from ignoring ambiguity (model uncertainty). Similar to Branger and Larsen (2013), Aït-Sahalia and Matthys (2019), and Jin et al. (2021), we measure this welfare loss in terms of the percentage of wealth loss when investors choose a suboptimal portfolio. Specifically, we must compare expected utility obtained from following the optimal robust portfolio choice with that obtained from following an alternative suboptimal portfolio choice. Expected utility associated with an arbitrary investment strategy $\pi$ is given by

32 $$\begin{aligned} J^{\pi } (t,w_t, v_t)= \inf _{u^S, u^V, u^N} {\mathbb{E}}^{{\mathbb{P}}^{u}}_{t} \left[ U (W_T) +\int _t^T \Psi (s,w, v) ds \right] , \end{aligned}$$

where $\Psi (t,w, v)$ is given by Eq. (7). Note that $u^S$, $u^V$, and $u^N$ depend on portfolio choice.

We solve investor’s optimization problem using the principle of optimal stochastic control, which leads to the following Hamilton-Jacobi-Bellman (HJB) equation for the value function $J^{\pi }$

33 $$\begin{aligned} \begin{aligned} 0=&\inf _{u^S, u^V, u^N} J^{\pi }_t + r w J^{\pi }_w + \pi (\eta v - \mu \lambda v - \sqrt{v} u^S ) w J^{\pi }_w + \frac{1}{2} v w^2 \pi ^2 J^{\pi }_{ww} + \frac{1}{2} \sigma ^2 v J^{\pi }_{vv} \\&\quad + \Psi + ( \alpha - \beta v -\kappa \lambda v - \sigma \rho \sqrt{v} u^S_t -\sigma \sqrt{1-\rho ^2} \sqrt{v} u^V_t )J^{\pi } _v \\&\quad + \rho \pi \sigma v w J^{\pi }_{wv} + \lambda v u^N {\mathbb{E}} \left[ J^{\pi } (t, w(1+\pi X),v +Y) - J^{\pi } \right] . \end{aligned} \end{aligned}$$

In what follows, we derive the welfare loss incurred when an investor chooses a portfolio while ignoring model uncertainty; thus, the investor chooses a suboptimal portfolio. Specifically, they follow the portfolio decision from Proposition 2 with $\theta _S=0, \theta _V=0,$ and $\theta _N=0$, i.e, $\pi ^{NU}=\pi (\theta _S=0, \theta _V=0, \theta _N=0)$. The following proposition gives an analytical solution for the suboptimal portfolio.

#### Proposition 4

The optimal value function of an investor who ignores model uncertainty is given by

34 $$\begin{aligned} J^{\pi }(t,w,v)=\frac{w^{1-\gamma }}{1-\gamma } e^{A^{\pi }(t)+B^{\pi }(t)v}. \end{aligned}$$

The worst-case measure is given by

35 $$\begin{aligned} u^{S} = & (\pi + \frac{1}{{1 - \gamma }}\sigma \rho B^{\pi } (t))\theta _{S} \sqrt v , \\ u^{V} = & \frac{1}{{1 - \gamma }}\sigma \sqrt {1 - \rho ^{2} } B^{\pi } (t)\theta _{V} \sqrt v , \\ u^{N} = & e^{{ - \frac{{\theta _{N} }}{{1 - \gamma }}\left( {{\mathbb{E}}[(1 + \pi X)^{{1 - \gamma }} e^{{B^{\pi } (t)Y}} ] - 1} \right)}} {\mkern 1mu} , \\ \end{aligned}$$

where $B^{\pi }(t)$ and $A^{\pi }(t)$ satisfy the following differential equation:

36 $$\begin{aligned} 0= & {} \frac{1}{1-\gamma } (B^\pi )^\prime (t) +\pi ( \eta - \mu \lambda )- \frac{1}{2} \pi ^{2} (\gamma + \theta _S) +\left( \frac{1 }{2(1-\gamma )} - \frac{ \rho ^2 \theta _S + (1-\rho ^2) \theta _V}{ 2(1-\gamma )^2}\right) \sigma ^2 (B^\pi )^2(t) \nonumber \\{} & {} \quad + \left( \rho \sigma \pi + \frac{\pi \sigma \rho \theta _S - (\beta +\kappa \lambda ) }{1-\gamma } \right) B^\pi (t) + \lambda \frac{1}{\theta _N} \left( 1- e^{ - \frac{\theta _N}{1-\gamma } \left( {\mathbb{E}} [ (1+\pi X)^{1-\gamma } e^{B^\pi (t) Y}]-1 \right) } \right) , \end{aligned}$$

and

37 $$\begin{aligned} \begin{aligned} 0= r (1-\gamma ) + (A^\pi )^\prime (t) + B^\pi (t) \alpha . \end{aligned} \end{aligned}$$

In addition, $$J^\pi (T,w,v) = \frac{w^{1-\gamma }_T}{1-\gamma }$$.

As mentioned above, we quantify welfare loss by the percentage of initial wealth that investors are willing to sacrifice to know the optimal robust portfolio. Hence, welfare loss *L* is defined by $J(t, w(1-L),v,\pi ^*)=J^\pi (t,w,v, \pi ),$ we have

38 $$\begin{aligned} \begin{aligned} L=1-e^{\frac{1}{1-\gamma }({A^{\pi } (t)-A(t)+(B^{\pi }(t)-B(t))v})}, \end{aligned} \end{aligned}$$

where $\pi = \pi ^{NU}$ is suboptimal portfolio choice and $\pi ^*$ is the optimal robust portfolio choice.

## Numerical results and discussions

In this section, we conduct numerical analysis to investigate the optimal portfolio choice problem under ambiguity in four steps: (1) confirming the effect of different ambiguity on Detection-error-probability (DEP), (2) identifying each ambiguity’s contribution over the optimal portfolio choice, (3) considering the effect of both price jump size and volatility jump size on the portfolio choice, (4) analyzing welfare loss in the incomplete market. To facilitate computation, we consider two special cases by taking the mean of $X_t$ and $Y_t$, following Liu et al. (2003) and Branger and Larsen (2013). We follow Pan (2002), Liu et al. (2003)^7^, and Aït-Sahalia et al. (2020)^8^ to set most parameter values. For the numerical analysis, we use parameter values given in the following Table 1.

**Table 1:** Parameter values for our numerical example

$\alpha$	*r*	$\beta$	$\sigma$	$\eta$	$\rho$	$\mu$	$\kappa$	$\lambda$
0.15	0.04	5.32	0.25	4.78	−0.62	−0.24	0.23	1.64

**Fig. 1:**
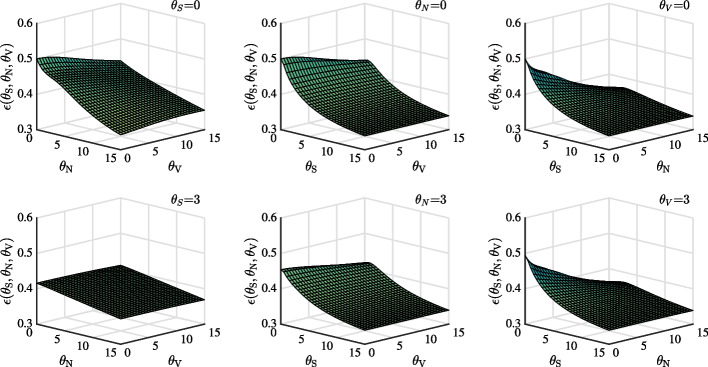
DEP in stock market as a function of ambiguity parameters $\theta _{S}$, $\theta _{V}$ and $\theta _{N}$. Panel (**a**), (**b**), and (**c**) set $\theta _{S}$, $\theta _{N}$, and $\theta _{V}$ as 0, respectively. By contrast, Panel (**d**), (**e**), and (**f**) set $\theta _{S}$, $\theta _{N}$, and $\theta _{V}$ as 3, respectively

In Fig. 1, we show DEP as a function of ambiguity aversion parameters and confirm that $\theta _{S}$ and $\theta _{N}$ mainly determine DEP in the case of no ambiguity as well as ambiguity. First, considering the no ambiguity case in Panel (a), (b), and (c), the larger the ambiguity aversion, the smaller the DEP, which is consistent with Branger and Larsen (2013). Since large ambiguity aversion allows investors to easily distinguish between the two models, we introduce ambiguity to review this effect. As seen in Panel (d), (e) and (f), when ambiguity is introduced, the DEP behaves in the same manner as in the previous case.

Second, we confirm that $\theta _{S}$ and $\theta _{N}$ mainly determine DEP. In Panel (a), when $\theta _{S}$=0, DEP evidently decreases with respect to $\theta _{N}$ but not with respect to $\theta _{V}$. In Panel (b) when $\theta _{N}$=0, it evidently decreases with respect to $\theta _{S}$, but not with respect to $\theta _{V}$. In Panel (c), when $\theta _{V}$=0, it substantially decreases with the increase in $\theta _{S}$ and $\theta _{N}$.

### Effect of the three sources of ambiguity on optimal portfolio choice

**Fig. 2:**
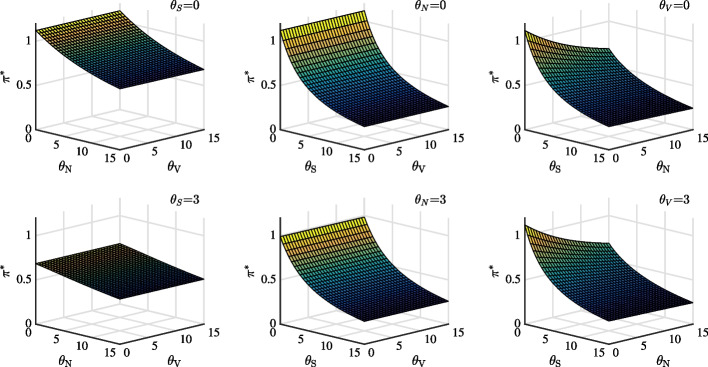
Optimal portfolio choice in stock market as a function of ambiguity aversion parameters $\theta _{S}$, $\theta _{V}$ and $\theta _{N}$. Panel (**a**), (**b**), and (**c**) set $\theta _{S}$, $\theta _{N}$ and $\theta _{V}$ as 0, respectively. By contrast, Panel (**d**), (**e**), and (**f**) set $\theta _{S}$, $\theta _{N}$, and $\theta _{V}$ as 3, respectively

Figure 2 shows the important role of $\theta _{S}$ and $\theta _{N}$ in determining the optimal choice. We choose parameters to ensure that the DEP is above 10%. From Panels (a) and (b), we can see that in cases without ambiguity, portfolio choice is relatively insensitive to $\theta _{V}$. Panel (c) indicates that when either $\theta _{S}$ or $\theta _{N}$ is relatively small, the exposure to the optimal portfolio decreases substantially with respect to $\theta _{S}$ or $\theta _{N}$. On the other hand, the exposure decreases slowly when either $\theta _{S}$ or $\theta _{N}$ is larger. Panel (d), (e), and (f) (case of ambiguity) show robust optimal portfolio choice result, compared to that in Panel (a), (b) and (c) (case of no ambiguity).

**Fig. 3:**
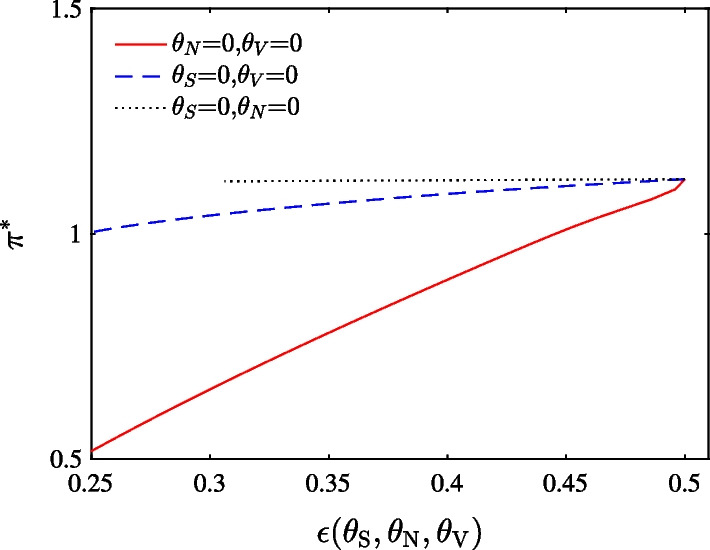
Optimal portfolio choice in stock market as a function of the DEP using different $\theta _{S}$, $\theta _{N}$ and $\theta _{V}$. The solid, dashed and dotted line represent change of ambiguity aversion with respect to price diffusion, jump and volatility diffusion while keeping the other two parameters as zero, respectively

To quantitatively identify which ambiguity is important, we further compare the effects of $\theta _{S}$, $\theta _{V}$, and $\theta _{N}$ on optimal portfolio choice as a function of DEP in Fig. 3. We begin with a DEP of 0.5, which corresponds to the case without ambiguity by setting each parameter as 0. When $\varepsilon _T(\theta _S, \theta _V, \theta _N)$ = 0.5, reference and alternative models are statistically indistinguishable. As seen from the Figure, the solid line lies below the dashed line. Therefore, optimal portfolio choice is more significantly affected by price diffusion ambiguity than by jump ambiguity. When DEP decreases from 0.5 to 0.3 due to an increase in $\theta _{S}$ (solid line), portfolio exposure reduces by almost 43%; however, reduction on account of $\theta _{N}$ (dashed line) is about 7%. Considering its whole range of portfolio choice, it is more significantly affected by $\theta _{S}$ than $\theta _{N}$. The smallest portfolio choice by price diffusion ambiguity is around 0.52, while that by jump ambiguity is around 1.01. Finally, varying volatility ambiguity $\theta _V$ (dotted line) does not influence portfolio choice when DEP decreases from 0.5 to about 0.31.

### Effects of price and volatility jump sizes under ambiguity on portfolio choice

**Fig. 4:**
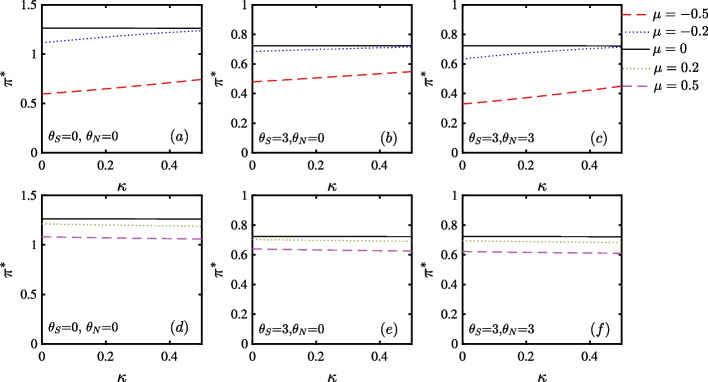
Effects of price and volatility jump sizes on portfolio choice in stock market. The top three panels show downward price jumps and no jump, while the bottom three panels show upward price jumps and no jump

In Fig. 4, we show effects of price and volatility jump sizes on portfolio choice under ambiguity. When volatility jump size is relatively small, the optimal portfolio choice for upward price jumps in Panel (d) is higher than that for downward price jumps in Panel (a). Second, according to the results in Panel (a) to (f), compared with the case of either downward or upward price jump, the investor usually chooses a greater portfolio choice when there is no price jump. However, the intercept of the vertical axis for the positive jump size in Panel (d) is higher than that for the negative price size in Panel (a). This asymmetry can be attributed to positive skewness. Here, the effects of jumps on return volatility overwhelms that of positive skewness, and thus, the investor takes a smaller portfolio portfolio even with a positive price jump size. Third, the investor’s portfolio increases with a large volatility jump size for downward jumps (negative price jump size). The effect is evident for $\mu = -0.2$ and less evident for $\mu =-0.5$. However, the investor’s portfolio is stable or decreases slowly with a large volatility jump size for upward price jumps (positive price jump size). The reasons are as follows. First, according to Liu et al. (2003) and Samuelson (1991), if an investor finds a large increase in volatility jump size after a negative (positive) price jump, the probability of earning successive negative (positive) returns is low. From a behavioral perspective, we can gauge that when the investor faces a run of successive negative returns or a negative jump in stock price, the probability of further runs of negative returns decreases. Therefore, they become more confident and take a larger stock position. This intuition is in line with Samuelson (1991). Second, in Panel (a) for a negative jump size, the investor is unwilling to sell the stock due to the disposition effect. The increase in portfolio choice during the downward cycle can be seen as “speculating,” for investors are more risk-tolerant and even speculate as they expect market reversal sooner and increase their portfolio choice. The larger the volatility jump size, the greater the inclination to speculate.

By contrast, in Panel (d) with large positive jump size, the investors slowly decreases their portfolio. Here, two counteracting effects account for this phenomenon: the “de-risking” effect (that explains that the ambiguity-averse investor ratchets down the portfolio) equals or marginally dominates the positive skewness effect (that explains that the investor is optimistic about the market and unwilling to decrease her portfolio)

Finally, we consider the ambiguity of price jump and volatility jump sizes in two steps. In Panels (b) and (e), adding $\theta _{S}$ brings about the robust result over the optimal portfolio choice. Since larger price jump sizes trigger larger volatility jumps, we add $\theta _{N}$ in Panels (c) and (f). Previous robust patterns still hold but with smaller portfolio choice, due to investor’s ambiguity aversion.

### Welfare analysis

**Fig. 5:**
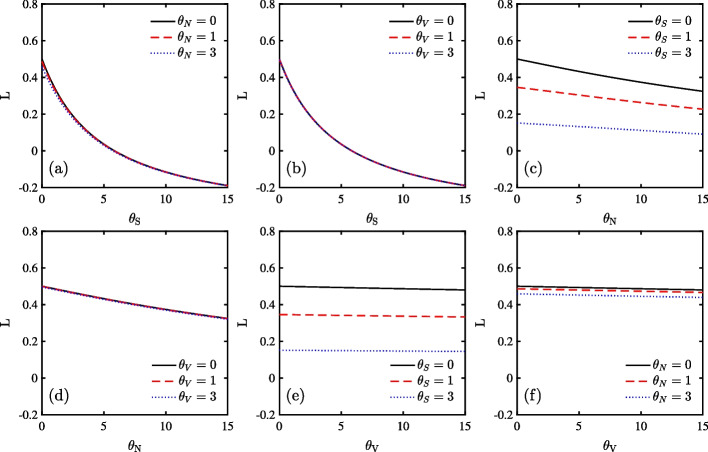
Expected utility loss in stock market as a function of the investor’s ambiguity parameters $\theta _{S}$, $\theta _{V}$ and $\theta _{N}$

In this subsection, we conduct welfare analysis in an incomplete market. Figure 5 plots expected welfare loss as a function of different ambiguity parameters of investors as three groups. The solid line, dashed line, and dotted line represent the different sources of ambiguity with 0, 1, and 3, respectively.

The first two panels depict the effect of price diffusion ambiguity on utility loss. In Panel (a), all lines are steeper at the initial stage and flatter when price diffusion ambiguity increases, indicating that when price diffusion ambiguity is very small, for example, in the range of 0 to 1 or 3, the welfare loss is relatively large. Therefore, investors should pay attention to price diffusion ambiguity when identifying such ambiguity during rare events. In Panel (b), the pronounced finding of Panel (a) also holds. Furthermore, the small gap between the three lines indicates that while different volatility ambiguity is secondary, price diffusion ambiguity plays a major role. Moreover, a close look at the utility loss change between Panels (a) and (b) indicates that expected utility loss driven by the combination of jump ambiguity and very small price diffusion ambiguity dominates the loss resulting from the combination of volatility ambiguity and very small price diffusion ambiguity. Next, Panels (c) and (d) depict the effect of jump ambiguity on utility loss. Both the magnitude and slope of utility loss are less than those in Panels (a) and (b). The utility loss changes dramatically from the initial case of $\theta _{S}$=0 (without price diffusion ambiguity) to the case of $\theta _{S}$=1 or 3 (small price diffusion ambiguity), confirming again the findings of Panel (a). Finally, the last two panels, Panels (e) and (f), depict the effect of volatility ambiguity on utility loss. In Panel (e), with increase in price diffusion ambiguity, welfare loss changes with different volatility ambiguities become larger. The flat lines again demonstrate that price diffusion ambiguity dominates jump ambiguity.

## Cryptocurrency market

In this section, we focus on Bitcoin for three reasons: first, Bitcoin is the largest cryptocurrency by market capitalization and is highly volatile. Second, Bitcoin volatility itself is time-varying and may jump significantly. Third, Bitcoin is exceptionally ambiguous. Based on this, we analyze dynamic portfolio choice of Bitcoin with uncertain rare event risk in the cryptocurrency market. Our model is inspired by two strands of literature: the first is represented by Hou et al. (2020) and Huang et al. (2022), who used the jump diffusion model of the stock market developed by Duffie et al. (2000) to study option pricing and leverage effect in the cryptocurrency market; the second is exemplified by Luo et al. (2021), who studied the existence of ambiguity aversion in the Bitcoin market and its impact on investor returns. It is natural to integrate the traditional stock market dynamic portfolio choice model and ambiguity in a single framework to analyze the investment decision problem in the cryptocurrency market under ambiguity.

**Fig. 6:**
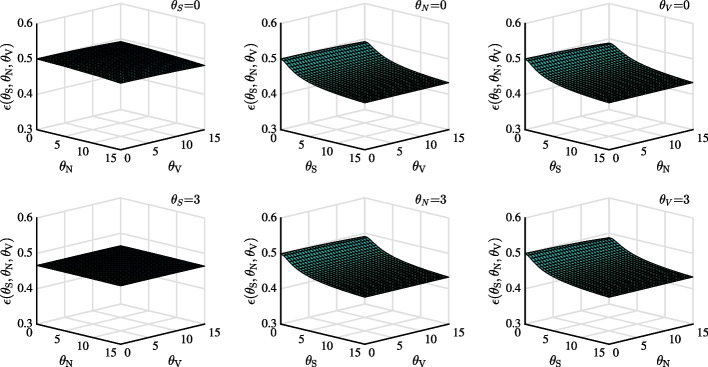
DEP in Bitcoin market as a function of ambiguity parameters $\theta _{S}$, $\theta _{V}$ and $\theta _{N}$. Panel (**a**), (**b**), and (**c**) set $\theta _{S}$, $\theta _{N}$, and $\theta _{V}$ as 0, respectively. By contrast, Panel (**d**), (**e**), and (**f**) set $\theta _{S}$, $\theta _{N}$, and $\theta _{V}$ as 3, respectively

**Fig. 7:**
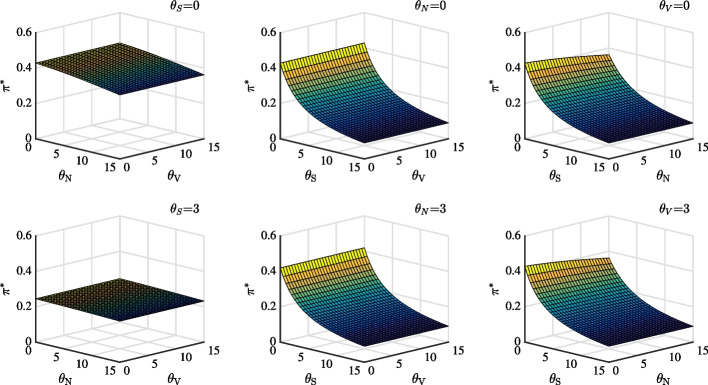
Optimal portfolio choice in Bitcoin market as a function of ambiguity aversion parameters $\theta _{S}$, $\theta _{V}$ and $\theta _{N}$. Panel (**a**), (**b**) and (**c**) set $\theta _{S}$, $\theta _{N}$ and $\theta _{V}$ as 0, respectively. By contrast, Panel (**d**), (**e**) and (**f**) set $\theta _{S}$, $\theta _{N}$ and $\theta _{V}$ as 3, respectively

**Fig. 8:**
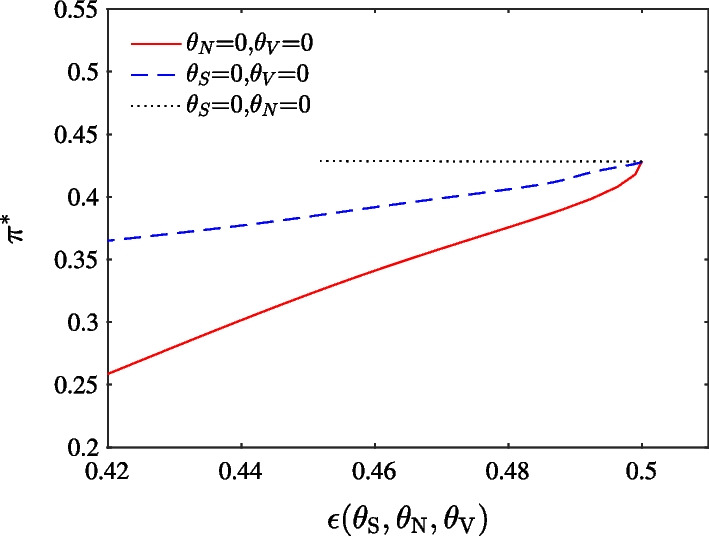
Optimal portfolio choice in Bitcoin market as a function of the DEP using different $\theta _{S}$, $\theta _{N}$ and $\theta _{V}$. The solid, dashed and dotted line represent change of ambiguity aversion with respect to price diffusion, jump and volatility diffusion while keeping the other two parameters as zero, respectively

Based on this idea, we replace the stock price and stochastic volatility in Eqs. (1) and (2) with the Bitcoin asset price and stochastic volatility. In an idea similar to the stock market model, we assume that investors in the Bitcoin market seek an optimal robust portfolio choice in a worst-case model. Thus, under the alternative measure, we solve the optimization problem (12) subject to constraints (4) and (5) to obtain the optimal robust portfolio choice and the worst-case measure. Furthermore, we can also obtain DEP and welfare loss in the Bitcoin market. We choose the Bitcoin parameter values given in Table 2; these are borrowed from Huang et al. (2022).^9^ DEP is presented in Fig. 6; portfolio choice concerning the ambiguity parameters is presented in Fig. 7; portfolio choice concerning DEP is presented in Fig. 8. We offer the following economic interpretation.

**Table 2:** Parameter values for our numerical example

$\alpha$	*r*	$\beta$	$\sigma$	$\eta$	$\rho$	$\mu$	$\kappa$	$\lambda$
0.14	0.04	4.05	0.32	1.80	0.65	−0.31	0.31	0.15

Figure 6 shows the similarity between the results obtained for the Bitcoin market and the traditional stock market model (in Fig. 1). In general, DEP is a monotonically decreasing function of ambiguity. We observe two striking features: first, the magnitude of change in DEP is smaller in the Bitcoin market than in the stock market. Second, the differences between the case without ambiguity parameter of the Bitcoin price and the case with the same parameter are relatively trivial. To be more specific, when we compare Figs. 1 and 6, the price diffusion ambiguity parameter dominates the other two parameters; this can be observed in Panels (a) and (d) of these two figures. In Panel (a) of Fig. 1, DEP exhibits a steep slope when jump ambiguity is taken into account, but a slowly decreasing DEP in panel (a) of Fig. 6. Moreover, the magnitude of DEP decreases in Panel (d) of Fig. 1 and is also larger than that in Panel (d) of Fig. 6. This is quite reasonable when we consider that rational investors will take into consideration uncertain rare events risk and make a conservative choice. This can also be explained from the information perspective: the traditional stock market has more liquidity and is more accessible to the investors. Thus, it contains more information when investors face ambiguity. Both financial knowledge and qualification in terms of the investor’s asset capacity make the Bitcoin market a novel area, or an area attracting a small volume of investors who prefer financial innovation to traditional markets. In other words, more information implies that investors have a higher chance of identifying the true model among the alternative models. The economic interpretation of the other four panels in Fig. 6 also has a similar underlying logic.

Compared with the traditional market, in the Bitcoin market, the change in optimal portfolio between the case without ambiguity and the case with ambiguity is substantially smaller. Figure 7 shows the important role of $\theta _{S}$ and $\theta _{N}$ in determining the optimal choice in the Bitcoin market. This result can be found in Fig. 2. Likewise, we also choose parameters to ensure that the DEP is above 10%. In cases without ambiguity, Panels (a) and (b) show that the portfolio choice is relatively stable with respect to $\theta _{V}$. Panel (c) shows that, when either $\theta _{S}$ or $\theta _{N}$ is relatively small, portfolio choice exposure decreases substantially with respect to $\theta _{S}$ or $\theta _{N}$ but the magnitude is smaller when price diffusion and jump ambiguity, $\theta _{S}$ or $\theta _{N}$, change from the case without ambiguity to the case with ambiguity. On the other hand, this choice decreases slowly when either $\theta _{S}$ or $\theta _{N}$ is larger, which is consistent with the pattern we have explained in Fig. 7. In cases with ambiguity, Panel (d), (e), and (f) (case of ambiguity) show the robust optimal portfolio choice result. In summary, investors reduce their portfolio choices when the degree of ambiguity aversion in the Bitcoin market is high.

**Fig. 9:**
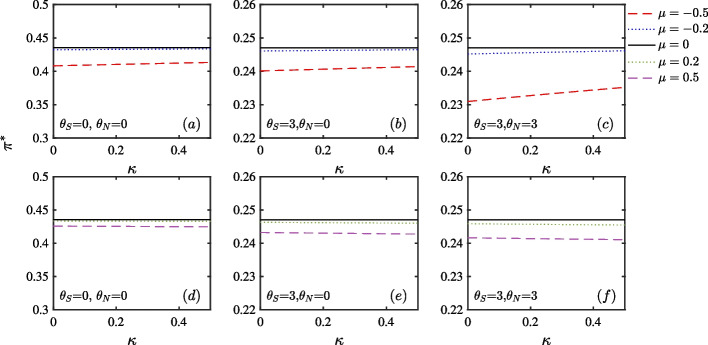
Effects of price and volatility jump sizes on portfolio choice in Bitcoin market. The top three panels show downward price jumps and no jump, while the bottom three panels show upward price jumps and no jump

In the similar analogy to Fig. 3, we compare the difference in impact of $\theta _{S}$, $\theta _{V}$, and $\theta _{N}$ on optimal portfolio choice in the Bitcoin market as a function of the DEP in Fig. 8 by quantitatively identifying the most important ambiguity. We begin with a DEP of 0.5, corresponding to the case without ambiguity, by setting each parameter as 0. When $\varepsilon _T(\theta _S, \theta _V, \theta _N)$ = 0.5, reference and alternative models are statistically indistinguishable. As we can see from Fig. 8, the solid line lies below the dashed line. Therefore, optimal portfolio choice is more significantly affected by price diffusion ambiguity than by jump ambiguity. When DEP decreases from 0.5 to 0.42 due to an increase in $\theta _{S}$ (solid line), portfolio choice reduces by about 40%, while the reduction due to $\theta _{N}$ is about 12% (dashed line). Here, the whole range of portfolio choice is more significantly affected by $\theta _{S}$ than by $\theta _{N}$. The smallest portfolio choice exposure by price diffusion ambiguity is around 0.26, while that by jump ambiguity is around 0.37. Volatility ambiguity $\theta _V$ (dotted line) does not influence the portfolio choice manifestly. These results show that cryptocurrency market echoes the traditional market.

In Fig. 9, we show effects of price and volatility jump sizes on portfolio choice under ambiguity in the Bitcoin market. We interpret the results from two aspects. On one hand, we find two similar results. First, the results for upward jumps are consistent with those in the traditional markets. Second, in comparison with the case of either a downward or upward price jump, the investor usually chooses a greater portfolio choice when there is no price jump, which is consistent with our previous findings. However, the intercept of the vertical axis for positive jump size in Panel (d) is almost the same as that for negative jump size in Panel (a). On the other hand, the investor’s choice increases with a large volatility jump size for very large downward jumps (negative price jump size). The effect is evident for $\mu = -0.5$. Such irrational investing behavior shows that in the Bitcoin market, even sophisticated investors speculate when exposed to extremely negative shocks.

**Fig. 10:**
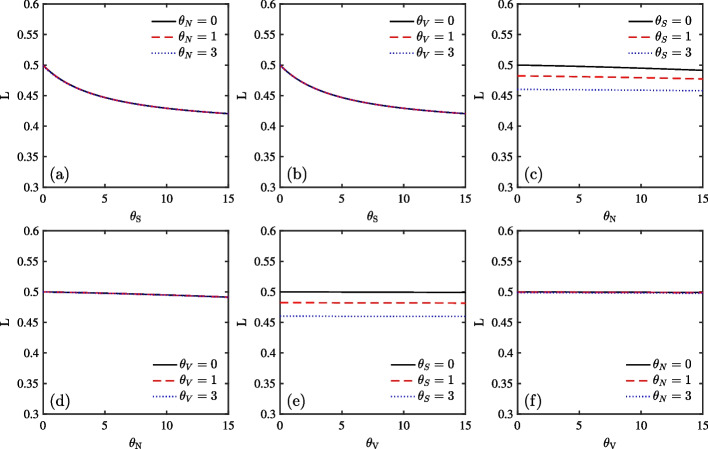
Expected utility loss in cryptocurrency market as a function of the investor’s ambiguity parameters $\theta _{S}$, $\theta _{V}$ and $\theta _{N}$

Next, we conduct a welfare analysis in an incomplete Bitcoin market. Figure 10 shows the expected welfare loss as a function of the investor’s different ambiguity parameters. The solid, dashed, and dotted lines represent the result from ambiguities 0, 1, and 3, respectively. Since all panels exhibit similar results in the traditional market (Fig. 5), we summarize welfare analysis in the Bitcoin market as follows. First, in both Panels (a) and (b), under the effect of price diffusion ambiguity, all lines exhibit steeper slopes initially and become flatter gradually. This suggests that investors take price diffusion ambiguity into consideration when making investment decisions. The small gap between the three different lines in Panel (b) indicates that different volatility ambiguity is secondary. Second, Panels (c) and (d) depict the effect of jump ambiguity on utility loss, where the slope of utility loss is less than that in Panels (a) and (b). The utility loss from different price diffusion ambiguity is larger than that from different volatility ambiguity. Finally, the last two panels depict the effect of volatility ambiguity on utility loss. Compared with the welfare loss of the volatility ambiguity over different jump diffusion ambiguity in Panel (f), that loss over different price diffusion ambiguity in Panel (e) changes more significantly.

## Conclusion

In this study, we integrated price diffusion, volatility diffusion, and jump ambiguity in both the traditional stock market and the emerging Bitcoin market into a single framework to examine the optimal portfolio choice during rare events. We first found that detection-error probability is mainly determined by price diffusion ambiguity and jump ambiguity. Then the optimal portfolio choice is more significantly affected by price diffusion ambiguity than by jump ambiguity, and the effect of volatility diffusion is trivial. Finally, we derived the optimal investment strategy during rare events. In particular, during negative price jumps, investors can “speculate” by increasing their exposure when volatility jump sizes are very large; during positive price jumps, they can “de-risk” by decreasing their exposure slowly. Our welfare analysis indicated that the investors in emerging economies should consider price diffusion ambiguity in the first instance.

Based on the study’s results, we offer some practical suggestions for investors in the stock market and the cryptocurrency market, facing high market ambiguity aversion. These are as follows: (1) they should decrease portfolio choice; (2) they should not increase leverage during periods of intense market volatility. Our investment advice to investors in cryptocurrency markets is based on the following two facts: on the one hand, the emerging cryptocurrency market is associated with low efficiency and high degree of asymmetric information, leading to few risk-hedging opportunities for investors; on the other hand, they tend to make less rational financial decisions due to less proficiency in financial expertise and risk consciousness. Therefore, compared with those in traditional markets, the investors in emerging economies should be more cautious about extremely high volatility in the market, seek more information, and practice value investing rather than speculation.

Regulators are advised to incorporate the following measure while designing policy: (1) educating investors so that they are abreast with the latest technology. This is urgent for investors in emerging economies as most investors in such economies are less informed, and noise traders comprise a large proportion; (2) developing diverse financial market products and derivatives to mitigate market incompleteness; (3) strengthening reasonable supervision, enhancing information transparency, and protecting the rights and interests of investors. Transparent information disclosure can benefit investors and encourage them to make rational decisions. The more effectively they protect the interests of the investors (through measures such as warning and punishing those violating the rules in the market), the more positive the feedback to investors.

## Data Availability

The numerical simulation analysis part of this study can be obtained from the corresponding author upon request.

## A Proof of Proposition 1

The proof of this proposition is inspired by Chiu and Wong (2018) and Mataramvura and Øksendal (2008)) and is divided into three steps to complete.

First, applying Ito’s formula to *J*(*t*, *w*, *v*) with respect to *W* and *V* under $\widetilde{{\mathbb{P}}}$ yields

A.1 $$\begin{aligned} {\mathbb{E}}^{\widetilde{{\mathbb{P}}}}_{t,w,v} \left[ J(\tau _N,W_{\tau _N},V_{\tau _N}) \right] = J(t,w,v)+{\mathbb{E}}^{\widetilde{{\mathbb{P}}}}_{t,w,v} \left[ \int _t^{\tau _N} {\mathcal{L}}^{u,\pi } J(s,W_s,V_s) ds \right] , \end{aligned}$$

where $\{\tau _N, N = 1, 2,\cdot \cdot \cdot \}$ is a localizing sequence of stopping times such that $\lim _{N \rightarrow \infty } \tau _N =T$. According to the ${\mathcal{L}}^{u,\pi ^*} J(t,w,v)+ \Psi (s,w,v,u,\pi ^*) \ge 0$, we have

A.2 $$\begin{aligned} J(t,w,v) \le {\mathbb{E}}^{\widetilde{{\mathbb{P}}}}_{t,w,v} \left[ U(W_{\tau _N}) + \int _t^{\tau _N} \Psi (s,W_s,V_s, u_s,\pi ^*) ds \right] . \end{aligned}$$

When $N \rightarrow \infty$, the equalities (*iv*) and (*v*) imply that

A.3 $$\begin{aligned} J(t,w,v) \le O^{u,\pi ^*} (t,w,v), \end{aligned}$$

since this holds for all $u \in U$ we deduce that

A.4 $$\begin{aligned} J(t,w,v) \le \inf _{u \in \Omega } O^{u,\pi } (t,w,v). \end{aligned}$$

Hence, we have

A.5 $$\begin{aligned} J(t,w,v) \le \sup _{\pi \in \Pi }\inf _{u \in \Omega } O^{u,\pi } (t,w,v). \end{aligned}$$

Next, we apply Eq. (A.1) to $u^*,\pi$, with $\pi \in \Pi$, and use ${\mathcal{L}}^{u^*,\pi } J(t,w,v)+ \Psi (s,w,v,u^*,\pi ) \le 0$ for all $v = V_t$ and $w = W_t$, we have

A.6 $$\begin{aligned} J(t,w,v) \ge {\mathbb{E}}^{\widetilde{{\mathbb{P}}}}_{t,w,v} \left[ U(W_{\tau _N}) + \int _t^{\tau _N} \Psi (s,W_s,V_s, u^*,\pi _s) ds \right] . \end{aligned}$$

When $N \rightarrow \infty$, the equalities (*iv*) and (*v*) imply that

A.7 $$\begin{aligned} J(t,w,v) \ge O^{u^*,\pi } (t,w,v) \ge \inf _{u \in U} O^{u,\pi } (t,w,v). \end{aligned}$$

Since this holds for all $\pi \in \Pi$, we deduce that

A.8 $$\begin{aligned} J(t,w,v) \ge \sup _{\pi \in \Pi } \inf _{u \in \Omega } O^{u,\pi } (t,w,v). \end{aligned}$$

Finally, we apply Eq. (A.1) to $u^*,\pi ^*$ and proceed as above. Then we have

A.9 $$\begin{aligned} J(t,w,v) = O^{u^*,\pi ^*} (t,w,v). \end{aligned}$$

Combining Eqs. (A.5), (A.8) and (A.9), we have

A.10 $$\begin{aligned} \inf _{u \in \Omega } \sup _{\pi \in \Pi } O^{u,\pi } (t,w,v) \le J(t,w,v) =O^{u^*,\pi ^*} (t,w,v) \le \sup _{\pi \in \Pi }\inf _{u \in \Omega } O^{u,\pi }(t,w,v). \end{aligned}$$

On the other hand, we have

A.11 $$\begin{aligned} \sup _{\pi \in \Pi } \inf _{u \in \Omega } O^{u,\pi } (t,w,v) \le \inf _{u \in \Omega } \sup _{\pi \in \Pi } O^{u,\pi }(t,w,v). \end{aligned}$$

By the inequalities (A.10) and (A.11), we have

A.12 $$\begin{aligned} \inf _{u \in \Omega } \sup _{\pi \in \Pi } O^{u,\pi } (t,w,v) = J(t,w,v) = \sup _{\pi \in \Pi }\inf _{u \in \Omega } O^{u,\pi }(t,w,v). \end{aligned}$$

## B Proof of Proposition 2

The first order condition with respect to the worst case measure $u^S$, $u^{V}$ and $u^{N}$ of the HJBI-equation (10) implies that

B.1 $$\begin{aligned} \begin{aligned} u^{S*} =&\pi \sqrt{v} w J_w \Psi ^S + \sigma \rho \sqrt{v} J_v \Psi ^S \,,\\ u^{V*}=&\sigma \sqrt{1-\rho ^2} \sqrt{v} J_v \Psi ^V \,, \\ u^{N*} =&e^{ - \Psi ^N {\mathbb{E}} \left[ J(t, w(1+\pi X), v +Y)-J \right] } \,. \end{aligned} \end{aligned}$$

Substituting (B.1) into HJBI-equation yields

B.2 $$\begin{aligned} \begin{aligned} 0=&\sup _{\pi } J_t + r w J_w + \pi (\eta - \mu \lambda )v w J_w - \frac{1}{2} \pi ^2 v w^2 J^2_w \Psi ^S - \pi \sigma \rho v w J_v J_w \Psi ^S \\&+( \alpha - \beta v -\kappa \lambda v ) J_v - \frac{1}{2} \sigma ^2 \rho ^2 v J^2_v \Psi ^S -\frac{1}{2} \sigma ^2 (1-\rho ^2) v J^2_v \Psi ^V + \frac{1}{2} v w^2 \pi ^2 J_{ww} \\&\ \ \ + \frac{1}{2} \sigma ^2 v J_{vv} + \rho \pi \sigma v w J_{wv} + \lambda v \frac{1}{\Psi ^N}(1-e^{ - \Psi ^N {\mathbb{E}} \left[ J(t, w(1+\pi X), v +Y)-J \right] } ) \,. \end{aligned} \end{aligned}$$

The first order conditions with respect to $\pi$, we can derive the optimal investment strategy follows

B.3 $$\begin{aligned} \begin{aligned} \pi ^* =&\frac{ (\eta -\mu \lambda ) J_w }{w (\Psi ^S J^2_w - J_{ww} )} +\frac{ \rho \sigma (J_{wv}- J_v J_w\Psi ^S)}{w (\Psi ^S J^2_w - J_{ww} )}+\frac{ \lambda {\mathbb{E}} \left[ XJ_{w} (t, w(1+\pi ^* X),v +Y) \right] }{w (\Psi ^S J^2_w - J_{ww} )}u^{N*}. \end{aligned} \end{aligned}$$

Suppose that solution is $J(t,w,v)=\frac{w^{1-\gamma }}{1-\gamma } e^{A(t)+B(t)v}$. By calculating the partial derivative, we have

B.4 $$\begin{aligned} J_{t} = & \frac{{w^{{1 - \gamma }} }}{{1 - \gamma }}e^{{A(t) + B(t)v}} (A^{\prime } (t) + B^{\prime } (t)v),J_{w} = w^{{ - \gamma }} e^{{A(t) + B(t)v}} ,\quad \\ J_{v} = & \frac{{w^{{1 - \gamma }} }}{{1 - \gamma }}e^{{A(t) + B(t)v}} B(t),J_{{ww}} = - \gamma w^{{ - \gamma - 1}} e^{{A(t) + B(t)v}} ,J_{{wv}} = w^{{ - \gamma }} e^{{A(t) + B(t)v}} B(t), \\ J_{{vv}} = & \frac{{w^{{1 - \gamma }} }}{{1 - \gamma }}e^{{A(t) + B(t)v}} B(t)^{2} . \\ \end{aligned}$$

We choose $\Psi ^i = \frac{\theta _i}{(1-\gamma )J(t,w,v)}, i=S,V,N$, where $\theta _S, \theta _V, {\theta _N} >0$. By simple calculation, we obtain

B.5 $$\begin{aligned} \begin{aligned} \frac{J_w (\eta -\mu \lambda ) }{w ( \Psi ^S J^2_w - J_{ww})} =\frac{\eta -\mu \lambda }{ \theta _S+\gamma }, \end{aligned} \end{aligned}$$

and

B.6 $$\begin{aligned} \begin{aligned} \frac{ \rho \sigma J_{wv}}{W ( \Psi ^S J^2_w - J_{ww})}=\frac{\rho \sigma B(t)}{ \theta _S+\gamma }. \end{aligned} \end{aligned}$$

Note that

B.7 $$\begin{aligned} \begin{aligned} {\mathbb{E}} \left[ J(t, w(1+\pi ^* X),v +Y)-J \right] = \frac{1}{1-\gamma } w^{1-\gamma } ( {\mathbb{E}} \left[ (1+\pi ^* X)^{1-\gamma } e^{B(t)Y}\right] -1) e^{A(t)+B(t)v}. \end{aligned} \end{aligned}$$

Substituting (13) into (B.3), we obtain the robust optimal robust portfolio choice (14). Plugging (13) in (B.1), we obtain the worst case measure. Substituting (B.4) - (B.7), $\Psi ^i, i=S,V,N$ and (14) into (B.2) and simplifying, we have

B.8 $$\begin{aligned} \begin{aligned} 0=&\frac{1}{1-\gamma } (A^\prime (t)+B^\prime (t) v) + r + \pi ^* (\eta - \mu \lambda ) v - \frac{1}{2} \pi ^{*2} \theta _S v +\frac{1}{1-\gamma } \pi ^* \sigma \rho \theta _S B(t) v + \alpha \frac{1}{1-\gamma } B(t) \\&- (\beta +\kappa \lambda ) \frac{1}{1-\gamma } B(t) v - \frac{ \sigma ^2 }{ 2(1-\gamma )^2} \rho ^2 B^2(t) \theta _S v - \frac{ \sigma ^2 }{ 2(1-\gamma )^2} (1-\rho ^2) B^2(t) \theta _V v \\&- \frac{1}{2} \pi ^{*2} \gamma v + \frac{1}{2(1-\gamma )} \sigma ^2 B^2(t) v + \rho \pi ^* \sigma B(t) v + \lambda v \frac{1}{\theta _N} (1- e^{ - \frac{\theta _N}{1-\gamma } ( {\mathbb{E}} \left[ (1+\pi ^* X)^{1-\gamma } e^{B(t)Y}\right] -1) })\,. \end{aligned} \end{aligned}$$

The right side of the above equation is an affine function in *v*. In order to make this equation hold for all *v*, then the constant term and the linear coefficient of *v* must be set to zero respectively, so we obtain the ordinary differential equation for *B*(*t*) and *A*(*t*) in (18) and (19). The terminal condition is $J(T,w,v)=U(w_T)$, where $U(w_T) = \frac{w^{1-\gamma }_T}{1-\gamma }$ is a power utility function.

## C Detection-error probabilities

Firstly, following Maenhout (2006) and Aït-Sahalia and Matthys (2019), by applying the Lévy’s general inversion, we obtain $\epsilon _T(\theta _S, \theta _V, \theta _N)$ is

$$\begin{aligned} \begin{aligned} \epsilon _T(\theta _S, \theta _V, \theta _N) = \frac{1}{2} - \frac{1}{2 \pi } \int _0^\infty \left\{ \Re \left( \frac{\phi _{\widetilde{{\mathbb{P}}}}(\omega , 0, T)}{i \omega } \right) -\Re \left( \frac{\phi _{\mathbb{P}}(\omega , 0, T)}{i \omega } \right) \right\} d \omega . \end{aligned} \end{aligned}$$

By using Feynman-Kac theorem, we solve two partial differential difference equations (PDDE) for $\phi _{\mathbb{P}}(\omega , t, T)$ and $\phi _{\widetilde{\mathbb{P}}}(\omega ,t,T)$ satisfying the corresponding boundary conditions and thus compute the $\phi _{\mathbb{P}}(\omega ,T,t)$ and $\phi _{\widetilde{\mathbb{P}}}(\omega ,t,T)$.

Secondly, define the Fourier transforms of the conditional expectation

$$\begin{aligned} \phi _{\mathbb{P}}(\omega ,t,T)= & {} {\mathbb{E}}{^{\mathbb{P}}}[e^{i \omega \xi _{T}}|{\mathscr {F}}_t ]= {\mathbb{E}}{^\mathbb{P}}[{\Lambda ^{i \omega }_{T} }|{\mathscr {F}}_t ]\,, \\ \phi _{\widetilde{\mathbb{P}}}(\omega ,t,T)= & {} {\mathbb{E}}^{\widetilde{\mathbb{P}}} [e^{i \omega \xi _{T}}|{\mathscr {F}}_t ]= {\mathbb{E}}^{\widetilde{\mathbb{P}}} [{\Lambda ^{i \omega }_{T} }|{\mathscr {F}}_t ] = {\mathbb{E}}{^\mathbb{P}}[e^{i \omega \xi _{T}} e^{\xi _{T}}|{\mathscr {F}}_t ]= {\mathbb{E}}{^\mathbb{P}}[{\Lambda ^{i \omega +1}_{T} }|{\mathscr {F}}_t ], \end{aligned}$$

which $\omega$ being the usual transforms variable, $i=\sqrt{-1}$, and $\xi _{t} \equiv \ln \Lambda _{t}.$ Let $\varvec{u}=[u^S, u^V]^T$ and $\varvec{\sigma } = [\rho \sigma \sqrt{V}, \sigma \sqrt{V} \sqrt{1-\rho ^2}]^T.$

Thirdly, by applying Feynman-Kac theorem, $\phi _{\mathbb{P}}(\omega , t, T)$ and $\phi _{\widetilde{\mathbb{P}}}(\omega , t, T)$ are given by the following differential equation

C.1 $$\begin{aligned} \begin{aligned} 0 =&\frac{ \partial \phi _{\mathbb{P}} }{\partial t} + (\alpha - \beta V - \kappa \lambda V) \frac{ \partial \phi _{\mathbb{P}} }{\partial V} -\frac{ \partial \phi _{\mathbb{P}} }{\partial \Lambda } \Lambda (u^N -1 )\lambda V +\frac{1}{2} \frac{ \partial ^2 \phi _{\mathbb{P}} }{\partial \Lambda ^2} \Lambda ^2 \Vert \varvec{u} \Vert ^2 \\&+\frac{1}{2} \sigma ^2 V \frac{ \partial ^2 \phi _{\mathbb{P}} }{\partial V^2} - \frac{ \partial ^2 \phi _{\mathbb{P}} }{\partial \Lambda \partial V} \Lambda \varvec{u} \varvec{\sigma }^T + \lambda V {\mathbb{E}} \left[ \phi _{\mathbb{P}} ( \omega , t, T)-\phi _{\mathbb{P}} (\omega , t-, T) \right] , \end{aligned} \end{aligned}$$

C.2 $$\begin{aligned} \begin{aligned} 0 =&\frac{ \partial \phi _{\widetilde{\mathbb{P}}} }{\partial t} +(\alpha - \beta V - \kappa \lambda V ) \frac{ \partial \phi _{\widetilde{\mathbb{P}}} }{\partial V} -\frac{ \partial \phi _{\widetilde{\mathbb{P}}} }{\partial \Lambda } \Lambda (u^N -1 )\lambda V +\frac{1}{2} \frac{ \partial ^2 \phi _{\widetilde{\mathbb{P}}} }{\partial \Lambda ^2} \Lambda ^2 \Vert \varvec{u} \Vert ^2 \\&+\frac{1}{2} \sigma ^2 V \frac{ \partial ^2 \phi _{\widetilde{\mathbb{P}}}}{\partial V^2} - \frac{ \partial ^2 \phi _{\widetilde{\mathbb{P}}} }{\partial \Lambda \partial V} \Lambda \varvec{u} \varvec{\sigma }^T + \lambda V {\mathbb{E}} \left[ \phi _{\widetilde{\mathbb{P}}} ( \omega , t, T)-\phi _{\widetilde{\mathbb{P}}} (\omega , t-, T) \right] , \end{aligned} \end{aligned}$$

with the boundary condition $\phi _{\mathbb{P}}(\omega ,T,T) = \Lambda ^{i \omega }_{T}$ and $\phi _{\widetilde{\mathbb{P}}}(\omega ,T,T) = \Lambda ^{i \omega +1}_{T}.$

Finally, we will calculate the differential equation. Let $p^S \equiv \frac{u^S}{\sqrt{V}}$, $p^V \equiv \frac{u^V}{\sqrt{V}}$. Suppose that solution is $\phi _{\mathbb{P}}(\omega , T,t)=\Lambda ^{i \omega }_{t}e^{D(t)+E(t)V}$. Substituting the solution into (C.1) yields

$$\begin{aligned} 0= & {} D'(t) + \alpha E(t)\,, \\ 0= & {} E'(t) - (\kappa \lambda + \beta ) E(t) - i \omega (u^N -1 )\lambda + \frac{1}{2} i \omega (i \omega -1) ((p^S)^2 +(p^V)^2) +\frac{1}{2} \sigma ^2 E^2(t) \\{} & {} - i \omega (\sigma \sqrt{1-\rho ^2} p^V +\rho \sigma p^S ) E(t) + \lambda {\mathbb{E}} \left[ (u^N)^{i \omega } e^{E(t)Y}-1 \right] , \end{aligned}$$

subject to $$D(T)=E(T)=0.$$

Likewise, suppose that solution is $\phi _{\widetilde{{\mathbb{P}}}}(\omega ,T,t)=\Lambda ^{i \omega +1}_{t}e^{F(t)+H(t)V}$. Plugging in (C.2) yields

$$\begin{aligned} 0= & {} F'(t) + \alpha H(t)\,, \\ 0= & {} H'(t) - (\kappa \lambda + \beta ) H(t) - (i \omega +1) (u^N -1 )\lambda + \frac{1}{2} i \omega (i \omega +1) ((p^S)^2 +(p^V)^2) \\{} & {} +\frac{1}{2} \sigma ^2 H^2(t)- (i \omega +1) (\sigma \sqrt{1-\rho ^2} p^V +\rho \sigma p^S ) H(t) + \lambda {\mathbb{E}} \left[ (u^N)^{i \omega +1} e^{H(t)Y}-1 \right] , \end{aligned}$$

subject to $$F(T)=H(T)=0.$$

## D Proof of Proposition 4

To obtain the suboptimal investment strategy, we solve the HJB equation (33). Solving the Minimization problem, the first order condition with respect to the worst case measure $u^S$, $u^{V}$ and $u^{N}$ of the HJB-equation (33) implies that

D.1 $$\begin{aligned} \begin{aligned} u^{S} =&\pi \sqrt{v} w J^\pi _w \Psi ^S + \sigma \rho \sqrt{v} J^\pi _v \Psi ^S \,,\\ u^{V}=&\sigma \sqrt{1-\rho ^2} \sqrt{v} J^\pi _v \Psi ^V \,, \\ u^{N} =&e^{ - \Psi ^N {\mathbb{E}} \left[ J^\pi (t, w(1+\pi X), v +Y)-J^\pi \right] } \,. \end{aligned} \end{aligned}$$

Substituting (D.1) into the HJB equation yields

D.2 $$\begin{aligned} \begin{aligned} 0=&J^\pi _t + r w J^\pi _w + \pi (\eta - \mu \lambda )v w J^\pi _w - \frac{1}{2} \pi ^2 v w^2 (J^\pi _w)^2 \Psi ^S - \pi \sigma \rho v w J^\pi _v J^\pi _w \Psi ^S \\&+( \alpha - \beta v-\kappa \lambda v )J^\pi _v - \frac{1}{2} \sigma ^2 \rho ^2 v (J^\pi _v)^2 \Psi ^S -\frac{1}{2} \sigma ^2 (1-\rho ^2) v (J^\pi _v)^2 \Psi ^V + \frac{1}{2} v w^2 \pi ^2 J^\pi _{ww} \\&+ \frac{1}{2} \sigma ^2 v J^\pi _{vv} + \rho \pi \sigma v w J^\pi _{wv} + \lambda v \frac{1}{\Psi ^N}(1-e^{ - \Psi ^N {\mathbb{E}} \left[ J^\pi (t, w(1+\pi X),v +Y) - J^\pi \right] } ) \,. \end{aligned} \end{aligned}$$

Suppose that solution is $J^\pi (t,w,v)=\frac{w^{1-\gamma }}{1-\gamma } e^{A^\pi (t)+B^\pi (t)v}$. By calculating the partial derivative have

D.3 $$\begin{gathered} J_{t}^{\pi } = \frac{{w^{{1 - \gamma }} }}{{1 - \gamma }}e^{{A^{\pi } (t) + B^{\pi } (t)v}} ((A^{\pi } )^{\prime } (t) + (B^{\pi } )^{\prime } (t)v), \hfill \\ J_{w}^{\pi } = W^{{ - \gamma }} e^{{A^{\pi } (t) + B^{\pi } (t)v}} , \hfill \\ J_{v}^{\pi } = \frac{{w^{{1 - \gamma }} }}{{1 - \gamma }}e^{{A^{\pi } (t) + B^{\pi } (t)v}} B^{\pi } (t),J_{{ww}}^{\pi } = - \gamma w^{{ - \gamma - 1}} e^{{A^{\pi } (t) + B^{\pi } (t)v}} ,J_{{wv}}^{\pi } = w^{{ - \gamma }} e^{{A^{\pi } (t) + B^{\pi } (t)v}} B^{\pi } (t),J_{{vv}}^{\pi } = \frac{{w^{{1 - \gamma }} }}{{1 - \gamma }}e^{{A^{\pi } (t) + B^{\pi } (t)v}} (B^{\pi } )^{2} (t). \hfill \\ \end{gathered}$$

We choose $\Psi ^i = \frac{\theta _i}{(1-\gamma ) J^\pi (W,V,t)}, i=S,V,N$, where $\theta _S, \theta _V, {\theta _N} >0$. By simple calculation, we obtain

D.4 $$\begin{aligned} \begin{aligned} \frac{J^\pi _w (\eta -\mu \lambda ) }{w ( \Psi ^S (J^\pi _w)^2 - J^\pi _{ww})} =\frac{\eta -\mu \lambda }{ \theta _S+\gamma }, \end{aligned} \end{aligned}$$

and

D.5 $$\begin{aligned} \begin{aligned} \frac{ \rho \sigma J^\pi _{wv}}{w ( \Psi ^S (J^\pi _w)^2 - J^\pi _{ww})} = \frac{\rho \sigma B^\pi (t)}{ \theta _S+\gamma }. \end{aligned} \end{aligned}$$

Note that

$$\begin{aligned} J^\pi (t, w(1+\pi X),v +Y) = \frac{1}{1-\gamma } w^{1-\gamma } (1+\pi X)^{1-\gamma }e^{A^\pi (t)+B^\pi (t)(v+Y)}. \end{aligned}$$

By simple calculation, we obtain

D.6 $$\begin{aligned} \begin{aligned} {\mathbb{E}} \left[ J^\pi (t, w(1+\pi X),v +Y) - J^\pi \right] = \frac{e^{A^\pi (t)+B^\pi (t)v}}{1-\gamma } w^{1-\gamma } ( {\mathbb{E}} \left[ (1+\pi X)^{1-\gamma } e^{B^\pi (t)Y}\right] -1). \end{aligned} \end{aligned}$$

Substituting (D.3)–(D.6), $\Psi ^i, i=S,V, N$ into (D.2), and simplify, we have

$$\begin{aligned} \begin{aligned} 0=&\frac{1}{1-\gamma } ((A^\pi )^\prime (t)+(B^\pi )^\prime (t) v) + r + \pi (\eta - \mu \lambda ) v- \frac{1}{2} \pi ^{2} \theta _S v +\frac{1}{1-\gamma } \pi \sigma \rho \theta _S B^\pi (t) v +\alpha \frac{1}{1-\gamma } B^\pi (t) \\&- (\beta +\kappa \lambda ) \frac{1}{1-\gamma } B^\pi (t) v - \frac{ \sigma ^2 }{ 2(1-\gamma )^2} \rho ^2 (B^\pi )^2(t) \theta _S v - \frac{ \sigma ^2 }{ 2(1-\gamma )^2} (1-\rho ^2) (B^\pi )^2(t) \theta _V v \\&- \frac{1}{2} \pi ^{2} \gamma v + \frac{1}{2(1-\gamma )} \sigma ^2 (B^\pi )^2(t) v + \rho \pi \sigma B^\pi (t) v + \lambda v \frac{1}{\theta _N} (1- e^{ - \frac{\theta _N}{1-\gamma } ( {\mathbb{E}} \left[ (1+\pi X)^{1-\gamma } e^{B^\pi (t)Y}\right] -1) })\,. \end{aligned} \end{aligned}$$

The right side of the above equation is an affine function in *v*. In order to make this equation hold for all *v*, then the constant term and the linear coefficient of *v* must be set to zero respectively, so we obtain the ordinary differential equation for $B^\pi (t)$ and $A^\pi (t)$ in (36) and (37). The terminal condition is $J(T,w,v)=U(w_T)$, where $U(w_T) = \frac{w^{1-\gamma }_T}{1-\gamma }$ is a power utility function.

The welfare loss *L* is defined by $J(t,w(1-L),v,T,\pi ^*) = J^\pi (t,w,v,t,T,\pi ),$ we have

D.7 $$\begin{aligned} \begin{aligned} L=1-e^{\frac{1}{1-\gamma }({A^{\pi } (t)-A(t)+(B^{\pi }(t)-B(t))v})}. \end{aligned} \end{aligned}$$

## Abbreviations

DEP: Detection-error probability
HJBI: Hamilton–Jacobi–Bellman–Isaacs
LTCM: Long term capital market

## Footnotes

1 Due to the financial friction, regulation and technology, financial market in both emerging economies and most developed ones around the world, including China with more than 180 million individual investors in 2021, can be classified as the incomplete market.

2 https://www.cnbc.com/2021/05/23/robert-shiller-sees-wild-west-in-housing-stock-and-crypto-markets.html.

3 Anderson et al. (2003) provide this powerful statistical tool used in Maenhout (2006).

4 Both Barber and Odean (2013) and Barberis and Thaler (2003) give comprehensive survey on the effect.

5 Shefrin and Statman (1985) found that investors are reluctant to sell asset at a loss.

6 Wen et al. (2019) find more information will benefit the investors to reduce the stock crash risk.

7 They accessed the monthly return series for U.S. stocks during the 1802 to 1925 period in Schwert (1990) and appended the CRSP monthly value-weighted index returns for the 1926 to 2000 period spanning nearly 200 years.

8 They analyzed daily data variance swap rates and S &P500 returns for the 1996 to 2010 period.

9 They accessed daily price data for cryptocurrency from coinmarketcap.com during 2017-2021.
